# Pleistocene isolation caused by sea-level fluctuations shaped genetic characterization of *Pampus minor* over a large-scale geographical distribution

**DOI:** 10.3897/zookeys.969.52069

**Published:** 2020-08-17

**Authors:** Yuan Li, Cheng Liu, Longshan Lin, Yuanyuan Li, Jiaguang Xiao, Kar-Hoe Loh

**Affiliations:** 1 Third Institute of Oceanography, Ministry of Natural Resources, Xiamen 361005, China Ministry of Natural Resources Xiamen China; 2 College of Marine Sciences, Shanghai Ocean University, Shanghai 201306, China Shanghai Ocean University Shanghai China; 3 Institute of Ocean and Earth Sciences, University of Malaya, Kuala Lumpur 50603, Malaysia University of Malaya Kuala Lumpur Malaysia

**Keywords:** Cytochrome b, genetic diversity, genetic structure, South China Sea, southern lesser pomfret

## Abstract

The southern lesser pomfret (*Pampus minor*) is an economically important fish, and its numbers are declining because of overfishing and environmental pollution. In addition, owing to the similarities of its external morphological characteristics to other species in the genus *Pampus*, it is often mistaken for grey pomfret (*P. cinereus*) or silver pomfret (*P. argenteus*) juveniles. In this study, the genetic diversity and structure of 264 *P. minor* individuals from 11 populations in China and Malaysia coastal waters were evaluated for the first time, to the best of our knowledge, using mitochondrial cytochrome b fragments. The results showed that *P. minor* had moderate haplotype diversity and low nucleotide diversity. Furthermore, two divergent lineages were detected within the populations, but the phylogenetic structure corresponded imperfectly with geographical location; thus, the populations may have diverged in different glacial refugia during the Pleistocene low sea levels. Analysis of molecular variation (AMOVA) showed that genetic variation originated primarily from individuals within the population. Pairwise *F*_ST_ results showed significant differentiation between the Chinese and Malaysian populations. Except for the Xiamen population, which was classified as a marginal population, the genetic differentiation among the other Chinese populations was not significant. During the Late Pleistocene, *P. minor* experienced a population expansion event starting from the South China Sea refugium that expanded outward, and derivative populations quickly occupied and adapted to the new habitat. The results of this study will provide genetic information for the scientific conservation and management of *P. minor* resources.

## Introduction

Because of the rise in fishing pressure, habitat destruction, and global climate change, understanding the level of marine biological variation and its genetic structure is of crucial significance to the protection of marine biological resources and genetic diversity (Butchart et al. 2010). Current research on population genetics mainly includes detecting the level of genetic diversity and population genetic structure within different species, estimating the effective population size, and investigating the mechanisms underlying various evolutionary factors (Ellegren 2014).

*Pampus minor* Liu & Li, 1998 is an offshore warm-water pelagic fish classified under the class Actinopterygii, order Perciformes and family Stromateidae. It is a newly discovered species, distributed primarily south of the mid-southern East China Sea and along the coast of Southeast Asian countries (Li et al. 2019a). Owing to the similarities in the external characteristics of *Pampus* species and the small size of *P. minor* (adults generally do not exceed 150 mm), this species was consistently mistaken for grey pomfret (*P. cinereus*) or silver pomfret (*P. argenteus*) juveniles in early studies (Liu and Li 1998).

The region in which *P. minor* is distributed experienced a series of glacial-interglacial cycles in the Late Quaternary. During glacial periods, fluctuations in sea levels led to massive changes in the area and structure of marginal seas (Wang 1999), which transformed the Western Pacific Ocean into an ideal marine region for studying how glacial periods affected marine life. We postulate that during the Last Glacial Maximum (LGM), *P. minor* was also strongly affected by the Pleistocene glacial period. Thus, under the harsh environmental conditions of the glacial period, most of the individuals within its distribution range went extinct, and only a handful of isolated populations in glacial refugia (such as the South China Sea) survived. As the climate warmed during interglacial periods, sea levels rose, which led to population expansion; hence, the corresponding phylogeographic patterns and population genetic structure may be detected.

There have been few studies on *P. minor* thus far, which have only focused on morphology (Liu and Li 1998; Li et al. 2019a), population genetics (Li et al. 2019b), and phylogenetics (Cui et al. 2010; Guo et al. 2010; Li et al. 2019a). To the best of our knowledge, no basic research has been conducted on the status and distribution of *P. minor* fishery resources, and there have been no reports analyzing the large-scale genetic structure of its distribution range. Given the general decline in fishery resources, *P. minor* resources have also been shrinking, and there is a need to understand its genetic diversity, genetic structure, effective population size, and other population genetic characteristics. These parameters form the basis for formulating strategies for the effective protection and rational exploitation and utilization of marine fishery resources (Funk et al. 2012).

In this study, mitochondrial DNA sequences (cytochrome b, Cytb) were used to study the genetic diversity, genetic structure, and historical demography of 11 *P. minor* populations in China and Malaysia coastal waters. In addition, the effects of paleoclimatic, paleo-geological, marine geological, environmental and other factors on population formation, distribution and expansion routes, as well as genetic exchange, were revealed. This enabled us to investigate the mechanisms underlying the current phylogeographic patterns of this species, which can serve as a scientific reference for fishery management.

## Materials and methods

### Sample collection

**Figure 1.**
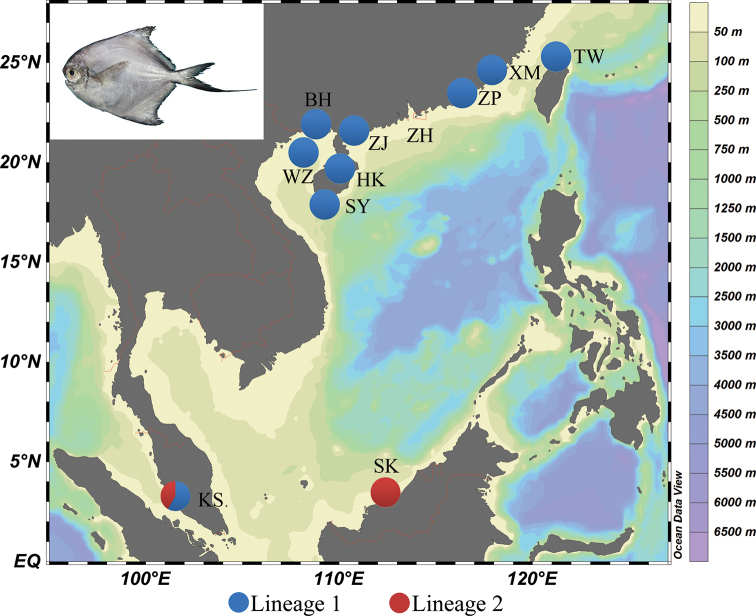
Sampling locations of *P. minor*. Populations are marked by abbreviations that correspond to Table 1.

Between May 2016 and December 2017, a total of 264 *P. minor* individuals from 11 geographical populations along the coasts of China (Xiamen, Zhangpu, Taiwan, Zhuhai, Zhanjiang, Beihai, Weizhou Island, Haikou, Sanya) and Malaysia (Kuala Selangor, Mukah) were collected (Fig. 1, Table 1). To ensure the accuracy of the taxonomy, the morphological identification of all specimens was based on Liu and Li (1998). A piece of back muscle tissue was frozen or preserved in 95% alcohol for molecular study.

**Table 1.** Information and molecular indices for *P. minor* based on mitochondrial DNA Cytb sequences.

Country	ID	Population	Number of individuals	Date	NH	NUH	*h*	π	*k*
China	XM	Xiamen Island	24	Apr. 2017	4	3	0.5391±0.1129	0.0007±0.0006	0.2917±0.2500
	ZP	Zhangpu	24	Apr. 2017	3	1	0.5942±0.0537	0.0016±0.0014	0.6667±0.5303
	TW	Taiwan	24	Oct. 2017	3	0	0.5072±0.0929	0.0013±0.0012	0.5507±0.4688
	ZH	Zhuhai	24	Dec. 2016	4	0	0.5326±0.1048	0.0015±0.0013	0.6015±0.4960
	ZJ	Zhanjiang	24	Dec. 2017	3	1	0.5399±0.0619	0.0014±0.0013	0.5725±0.4805
	BH	Beihai	24	Nov. 2016	5	1	0.6377±0.0606	0.0019±0.0016	0.7681±0.5824
	WZ	Weizhou Island	24	Nov. 2016	3	0	0.5543±0.0525	0.0014±0.0013	0.5906±0.4903
	HK	Haikou	24	Dec. 2016	5	1	0.4855±0.1129	0.0013±0.0012	0.5399±0.4629
	SY	Sanya	24	Dec. 2016	3	0	0.4891±0.0843	0.0012±0.0011	0.5145±0.4491
Malaysia	KS	Kuala Selangor	24	May 2016	7	3	0.6341±0.0973	0..49±0.0031	2.0109±1.1731
	SK	Mukah	24	May 2016	7	4	0.6087±0.1115	0.0038±0.0026	1.5652±0.9669
Total			264	–	22	14	0.6763±0.0189	0.0035±0.0023	1.4385±0.8794

Note: NH, number of haplotypes; NUH, number of unique haplotypes; *h*, haplotype diversity; π, nucleotide diversity; *k*, mean number of pairwise differences

### DNA extraction, amplification, and sequencing

Genomic DNA of *P. minor* was extracted from muscle tissue using a Qiagen DNeasy kit. The genomic DNA was assessed by electrophoresis with a 1.5% agarose gel and qualified samples were stored at 4 °C for PCR amplification. The mtDNA cytochrome b (Cytb) was amplified with the primers L14734: 5’-AACCACCGTTGTTATTCAACT-3’ (Inoue et al. 2001) and H15149: 5’-CTCAGAATGATATTTGTCCTCA-3’ (Ohdachi et al. 1997). All PCR reactions were carried out in a final mixture of 25 μL: 0.15 μL Taq polymerase, 2.5 μL 10× PCR buffer, 17.5 μL ultrapure water, 2 μL dNTPs, 1 μL of forward primer (5 μM), 1 μL of reverse primer (5 μM), and 1 μL of template DNA. PCR was carried out by initial denaturation step at 94 °C for 4 min, then followed by 32 cycles of denaturation at 94 °C for 30 sec, annealing at 50 °C for 30 sec, and extension at 72 °C for 30 sec, and plus a final extension at 72 °C for 10 min. After purification of the PCR products, both DNA strands were sequenced. The newly determined Cytb sequences were deposited in GenBank under the accession numbers (MT303974–MT303978, MF616364–MF616380).

### Data analysis

The Cytb sequences were aligned using the DNASTAR (Madison, WI, USA) software and manually edited. Haplotypes were defined based on sequence data without considering sites with gaps using DnaSP ver. 5.00 (Librado and Rozas 2009). Genetic diversity in each population was accessed as polymorphic sites, haplotype number, mean number of pairwise differences, haplotype diversity, and nucleotide diversity using ARLEQUIN version 3.5 (Excoffier et al. 2005). Analysis of molecular variation (AMOVA) in ARLEQUIN software was employed to investigate the genetic variation and test population structure. The MEGA 5.0 (Tamura et al. 2011) was applied to reconstructed the neighbor-joining (NJ) tree based on the genetic distance among haplotypes, and implemented with 1000 replicates. The relationships of haplotypes by unrooted minimum spanning tree (MST) was evaluated via the MINSPNET algorithm in ARLEQUIN software (Excoffier et al. 2005), and the MST topological structure was subsequently drawn by hand.

Both neutrality testing and mismatch distribution analysis were used to infer the historical demography expansions, as implemented in ARLEQUIN. Deviations from neutrality, significant negative values of Fu’s *F*s and Tajima’s *D* statistic, were evaluated to experience population growth and spatial range expansion. A molecular clock-based time estimate provided an approximate timeframe for evaluating phylogeographical hypotheses. Historical demographic expansions were further tested by nucleotide mismatch distribution, based on three parameters: *θ_0_*, *θ_1_* (*θ* before and after population growth), and *τ* (time since expansion, expressed in units of mutational time) (Rogers and Harpending 1992). The real-time since expansion was computed by the equation *τ*=2×*μ*×*t*, where *μ* is the mutation rate for the whole sequence and *t* is the time since expansion.

In the present study, a sequence divergence rate of 0.2×10^–7^/site/year (Avise 1998; Sun and Tang 2018) was applied to the Cytb sequences of *P. minor*. Bayesian skyline plots were created using BEAST v.8 (Drummond et al. 2012). However, a molecular clock-based time estimate provided an approximate time frame for evaluating phylogeographic hypotheses.

## Results

### Genetic diversity

A total of 264 sequences were obtained from the 11 *P. minor* populations. After manual alignment, a target fragment of 415 bp was obtained, of which there were 19 polymorphic sites, 12 singleton sites, seven parsimony informative sites, and no indels. A+T content (62.32%) was significantly higher than G+C content, thus showing an AT bias.

**Figure 2.**
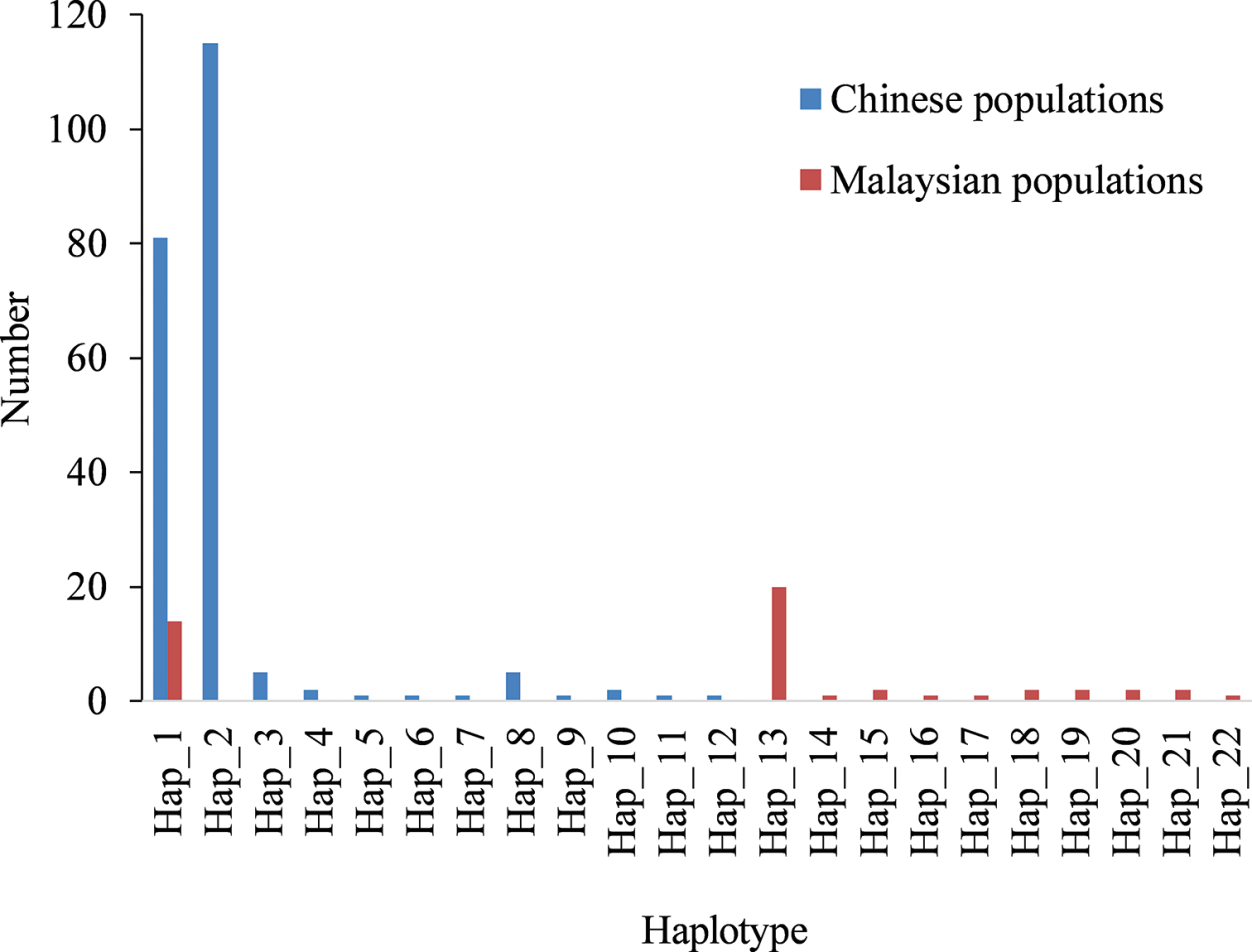
Composition and distribution of 22 Cytb haplotypes in the Chinese and Malaysian populations.

A total of 22 Cytb haplotypes were defined in the 264 individuals. The number of haplotypes in each population ranged from three to seven. The number of haplotypes shared by two or more populations was eight (36.4%). There were 14 unique haplotypes (63.6%), and seven populations had unique haplotypes, with the number ranging from one (ZP, ZJ, BH, HK) to four (SK) (Table 1). There were large differences in the composition and distribution of haplotypes between the Chinese and Malaysian populations, with only one haplotype shared between the two. Both the Chinese (7/12=58.3%) and Malaysian (7/11=63.6%) populations were dominated by unique haplotypes (Fig. 2). The haplotype with the highest frequency was Hap_2, consisting of 115 sequences. Hap_1, consisting of 95 sequences, was the only haplotype shared by the Chinese and Malaysian populations and may be an ancestral haplotype.

In general, the *P. minor* populations exhibited moderate haplotype diversity (0.6763 ± 0.0189) and low nucleotide diversity (0.0035 ± 0.0023). This phenomenon is usually due to bottleneck effects, resulting in population expansion or rapid population growth in small populations, accompanied by the generation of a large number of new mutations (Avise et al. 1984; Grant and Bowen 1998).

### Genetic structure

**Figure 3.**
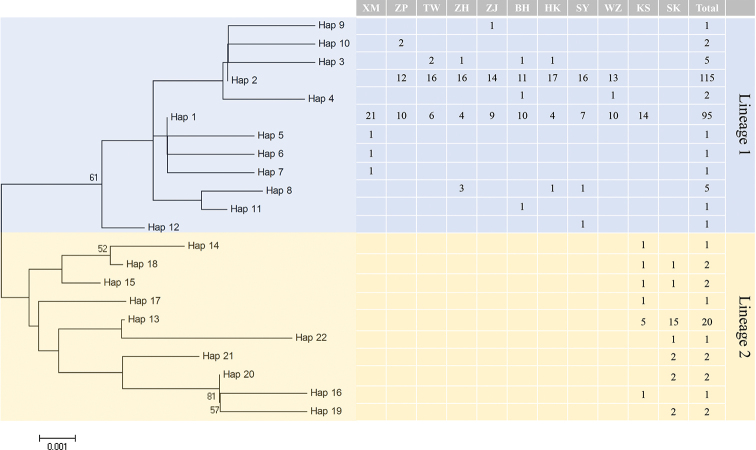
NJ tree and distribution of Cytb haplotypes among populations for *P. minor*. Bootstrap supports of > 50 in 1000 replicates are shown.

An NJ tree was constructed based on the 22 *P. minor*Cytb haplotypes. The results showed two divergent lineages detected within the populations but with low bootstrap values. The phylogenetic structure detected corresponded imperfectly to the geographical locations (Fig. 3). Lineage 1 was composed of 12 haplotypes (230 individuals), and lineage 2 was composed of ten haplotypes (34 individuals) (Fig. 3). Lineage 1 was composed of all the Chinese populations and some individuals from Malaysia KS, whereas lineage 2 was composed entirely of Malaysian populations.

**Figure 4.**
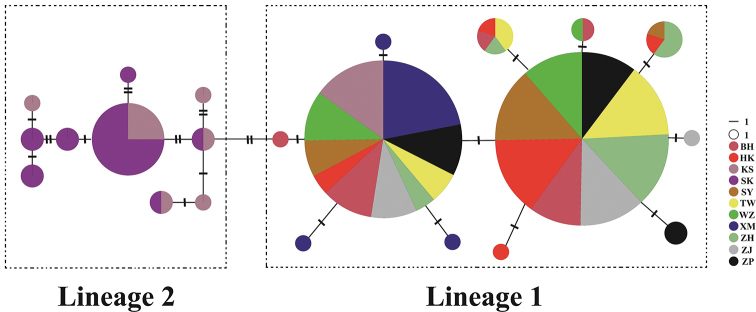
Unrooted minimum spanning tree showing the genetic relationships among the Cytb haplotypes of *P. minor*. Circle sizes are proportional to haplotype frequency. Perpendicular tick marks on the lines joining the haplotypes represent the number of nucleotide substitutions.

An unrooted MST was constructed based on the 22 Cytb haplotypes of the Chinese and Malaysian populations (Fig. 4). All sequences exhibited multiple primary haplotypes, and the other haplotypes were radially distributed around the primary haplotypes with obvious phylogenetic structures that corresponded to the Chinese and Malaysian populations.

Based on the TrN+G model, the net genetic distance between the two haplotype lineages was 0.006. Based on a mitochondrial Cytb sequence divergence rate of 2% per million years, the time of divergence between lineages 1 and 2 was approximately 300 thousand years ago (Kya).

**Figure 5.**
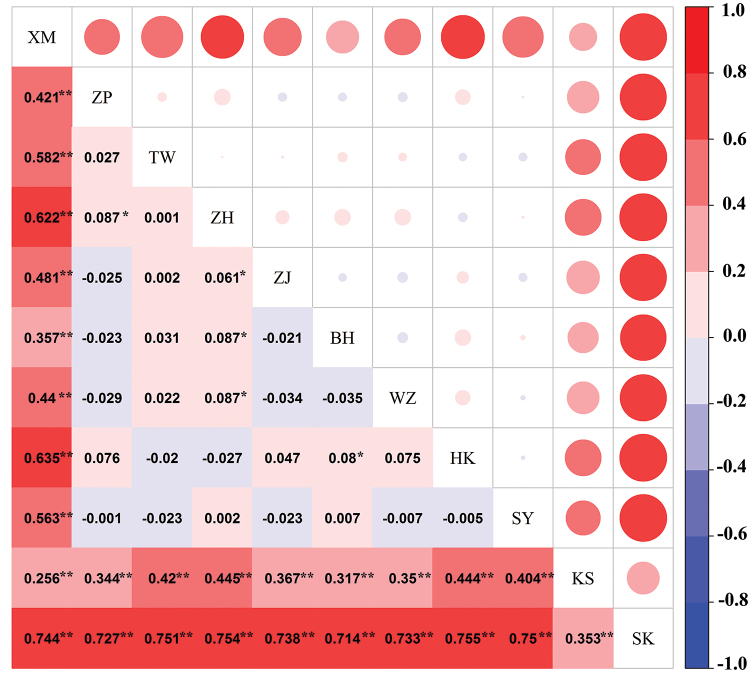
Matrix of pairwise *F*_ST_ values between 11 *P. minor* populations based on Cytb sequences. ^＊^ significant at *p* < 0.05 by the permutation test, ^＊＊^ extremely significant at *p* < 0.01 by the permutation test.

The fluctuation ranges of pairwise *F*_ST_ between populations were relatively large. The *F*_ST_ values between the Chinese and Malaysian populations were all above 0.25, and statistical tests indicated significance, thus showing very great differentiation (Wright 1965) (Fig. 5). The *F*_ST_ values between the Xiamen and other populations were relatively large, and differentiation was statistically significant. Except for the Xiamen population, the *F*_ST_ values between the Chinese populations were below 0.15, thus showing limited differentiation, and most of the statistical tests were not significant. Some *F*_ST_ values among the *P. minor* populations were still negative, suggesting that the level of differentiation within *P. minor* populations was greater than that among populations (Aris-Brosou and Excoffier 1996).

AMOVA was used to detect the genetic structure of the populations (Table 2). First, all *P. minor* populations were analyzed as one gene pool. The results showed significant genetic differentiation within each population (*Φ*_ST_ = 0.477, *p* < 0.01), accounting for 52.26% of the variation, whereas genetic differentiation among populations accounted for only 47.74% of the variation.

**Table 2.** AMOVA analysis of *P. minor* populations based on mitochondrial Cytb sequences.

Source of variation	Sum of squares	Percentage	Φ statistic	*p*
***One gene pool*** (XM, ZP, TW, ZH, ZJ, BH, WZ, HK, SY, KS and SK)				
Among populations	89.92	47.74	*Φ*_ST_=0.477	0.00
Within populations	99.25	52.26		
***Two gene pools*** (XM, ZP, TW, ZH, ZJ, BH, WZ, HK and SY) (KS and SK)				
Among groups	64.23	30.74	*Φ*_CT_=0.612	0.01
Among populations within groups	25.69	8.04	*Φ*_SC_=0.207	0.00
Within populations	99.25	61.23	*Φ*_ST_=0.693	0.00
***Four gene pools*** (TW) (XM, ZP, ZH, ZJ, BH and WZ) (HK and SY) (KS and SK)				
Among groups	67.08	40.30	*Φ*_CT_=0.403	0.03
Among populations within groups	22.83	13.95	*Φ*_SC_=0.234	0.00
Within populations	99.25	45.75	*Φ*_ST_=0.542	0.00
***Six gene pools*** (TW) (XM, ZP, ZH and ZJ) (BH and WZ) (HK and SY) (KS) (SK)				
Among groups	79.87	42.54	*Φ*_CT_=0.425	0.04
Among populations within groups	10.05	8.43	*Φ*_SC_=0.147	0.00
Within populations	99.25	49.03	*Φ*_ST_=0.510	0.00

To further confirm the genetic structure of the *P. minor* populations, the 11 populations were grouped into two, four, and six gene pools based on their geographic distribution. The results of all groupings showed that the genetic differentiation among groups was relatively large with statistical significance (*p* < 0.05), whereas genetic differentiation originating primarily within populations was highly significant (*p* < 0.01), and genetic differentiation among populations within groups was also significant (*p* < 0.01).

### Historical demography

**Figure 6.**
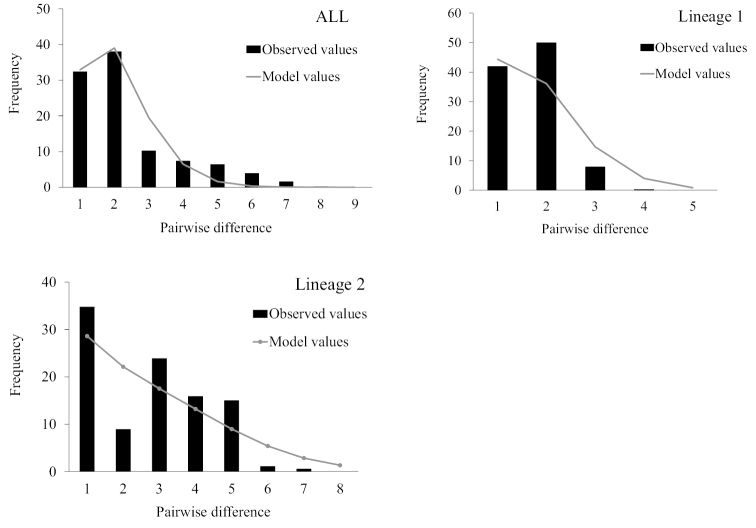
The expected mismatch distributions under a sudden expansion model (solid gray line) and the observed pairwise difference (black bars) of Cytb haplotypes of *P. minor*.

**Figure 7.**
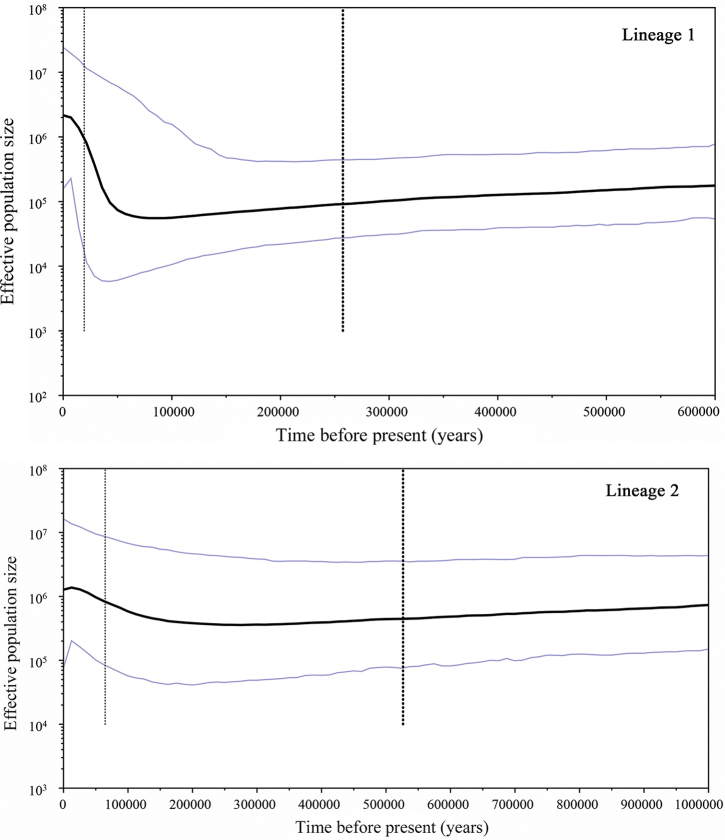
BSPs showing *N_ef_*T (*N_ef_* = effective population size; T = generation time) changes over time for *P. minor* based on Cytb sequences. The upper and lower limits of the blue line represent the 95% confidence intervals of highest posterior densities (HPD) analysis. The solid black line represents median estimates of *N_ef_*T.

Two haplotype lineages were detected in all Chinese and Malaysian populations with imperfect geographic lineage structures. Due to the significant differentiation among all populations, the historical demography of the two haplotype lineages was analyzed. The nucleotide mismatch distribution in all *P. minor* sequences was unimodal, and similar results were found in both lineages (Fig. 6). Neutrality test results showed that the Fu’s *F*_S_ tests for each lineage and the overall population yielded negative values, and were statistically significant (*p* < 0.05). Tajima’s *D* test for each lineage and the overall population yielded negative values, and were all statistically significant, except for lineage 2. In addition, the *SSD* and *HRI* test indices were not significant (*p* > 0.05) (Table 3), indicating that there was no significant deviation from the expected distribution under the population expansion model. Therefore, this can be used to analyze the historical demography of the *P. minor* populations, implying that this species has experienced population expansion events. Simultaneously, the Bayesian skyline plot (BSP) also indicated the same result (Fig. 7).

**Table 3.** Summary of molecular diversity, neutral test and goodness-of-fit test for *P. minor*.

	Number	NH	*h* ± SD	*л* ± SD	*k* ± SD	Tajima’s *D*		Fu’s *Fs*		Goodness-of-fit test				
						*D*	*p*	*Fs*	*p*	τ	*θ_0_*	*θ_1_*	*SSD*	*HRI*
Lineage 1	230	7	0.5807 ± 0.0177	0.0016 ± 0.0013	0.6643 ± 0.5131	-1.378	0.044	-7.647	0.003	0.836	0.011	83022	0.0258ns	0.1889ns
Lineage 2	34	7	0.6524 ± 0.0917	0.0042 ± 0.0028	1.7273 ± 1.0316	-0.905	0.189	-3.499	0.030	1.813	0.000	2.601	0.0321ns	0.1151ns
All	264	22	0.6763 ± 0.0189	0.0035 ± 0.0023	1.4385 ± 0.8794	-1.379	0.045	-12.923	0.001	1.000	0.000	99999	0.026ns	0.109ns

Note: NH, numbers of haplotypes; *h*, haplotype diversity; *л*, nucleotide diversity; *k*, average number of pairwise differences; ns, *p* > 0.05

The τ value of the nucleotide mismatch distribution provides a time point for estimating population expansion. The τ value of lineage 2 was 1.813 (95% CI: 0.059–5.600), which was larger than that of lineage 1 (0.836, 95% CI: 0.572–1.357) (Table 3). Based on a divergence rate of 0.2×10^–7^/site/year and τ, the population expansion time points of lineages 1 and 2 were estimated to be 101 and 218 Kya, respectively, which was during the Late Pleistocene. The ratio of *θ_1_* after expansion to *θ_0_* before expansion is infinite, indicating a sharp increase in the size of the effective maternal population of *P. minor* after population expansion. BSPs for lineage 1 and lineage 2 showed that both lineages have undergone the Late Pleistocene demographic expansion (Fig. 7), which started at different times. The effective population size of lineage 2 increased slowly, while the effective population size of lineage 1 increased sharply after the LGM.

## Discussion

Genetic diversity is the basis of both species and ecological diversity, while species and genetic diversity are both the basis of ecosystem diversity. Studies on the genetic diversity of species have attracted increasing attention from domestic and international researchers. The genetic diversity of a species directly affects its adaptation to the environment: the higher its level of diversity, the greater its potential for evolution and the stronger its adaptation to environmental changes, whereas the opposite implies the possibility of its deterioration or extinction (Rosel et al. 1995).

Compared to the levels of intraspecific genetic diversity of Cytb gene sequences in *Trachidermus fasciatus* (*h* = 0.97 ± 0.011) (Gao et al. 2013), *P. argenteus* (*h* = 0.775 ± 0.041) (Zhao et al. 2011a), *Anguilla mossambica* (0.691 ± 0.043) (Frankowski et al. 2020) and other fishes, the genetic diversity of Chinese and Malaysian *P. minor* was at a moderate level. From a historical evolutionary perspective, large population sizes, environmental heterogeneity and life-history traits that favor rapid population increases are the main reasons for maintaining high haplotype diversity in natural populations of marine fishes. *Pampus minor* is widely distributed in the mid-southern East China Sea, South China Sea, and the coastal regions of Southeast Asian countries, indicating that large population sizes may account for the relatively high levels of haplotype diversity observed in this study. However, little is known about these life-history traits for *P. minor*, and further study is needed to examine this correlation. In any case, the *P. minor* populations had moderate haplotype diversity and low nucleotide diversity. Fish populations with this type of diversity pattern may have experienced historical expansion events, and a population bottleneck followed by rapid population growth and accumulation of mutations (Grant and Bowen 1998).

Stepien (1999) suggests that the stable number of shelf fish over the long term and a large effective population are the causes of its high haplotype diversity. Although pomfret fishery resources have declined due to overfishing (Jin et al. 2005), the larger amount of fishery resources and the number of effective populations of *P. minor* compared to other economic fishes have helped maintain its moderate level of genetic diversity. In addition, *P. minor* is a widely distributed species and has a wide dietary preference. Its spawning grounds exhibit different characteristics according to the different marine regions in which it is distributed, and its habitat conditions are heterogeneous (Wu et al. 2012). These life-history traits and the environmental heterogeneity of *P. minor* can promote rapid population growth, implying that the natural selection pressure faced by the population is relatively small, which may have led to the increased accumulation of genetic mutations and a rich genetic diversity.

The genetic diversity distribution of a species is not only affected by past historical events, but also by current evolutionary forces (e.g., migration). The discontinuity of habitats and the instability of population changes can result in differentiation between species populations (Stepien 1999). Both the NJ tree and unrooted MST showed two divergent lineages of *P. minor*. AMOVA also showed that the genetic differentiation primarily originated within populations. The time of divergence of the two lineages was approximately 300 Kya, and the different glacial refugia during Pleistocene low sea levels may have caused the divergence of the two lineages. Many previous studies have shown that Pleistocene glaciations are important factors in the genetic differentiation of many marine organisms (Liu et al. 2007; Shen et al. 2011; Zhao et al. 2011a, b; Wu et al. 2012; Gao et al. 2013).

In the Late Quaternary, the global climate experienced a series of glacial-interglacial cycles. In the last 800 Kya, climate fluctuations mainly occurred at intervals of ~100 Kya (Lambeck et al. 2002). The fourth glacial period ended at about 420 Kya (Petit et al. 1999), which coincided with the time of divergence for the two lineages of *P. minor*. With the arrival of the fourth glacial period, the sea level fell by about 120~140 m, and *P. minor* populations may have become isolated in the South China Sea refugium. After the glacial period, as sea levels rose, *P. minor* populations in the refugium expanded toward the coasts of China and Malaysia. The NJ tree and haplotype network tree show that Hap_1 was the only shared haplotype between the Chinese and Malaysian populations, and accounted for a relatively large number of individuals. This indicated that it was the ancestral haplotype, thus further demonstrating that the *P. minor* populations originated from the same refugium.

The protein encoded by the mitochondrial Cytb gene acts as a subunit in complex V of the oxidative phosphorylation pathway (Saraste 1999). Studies have shown that non-synonymous mutations in the mitochondrial Cytb gene can affect the metabolism- and energy-related selective evolution of animals (Mishmar et al. 2003; da Fonseca et al. 2008; Xu et al. 2017). In marine fishes, different dimensions of natural selection between populations may be related to temperature adaptation and aerobic exercise associated with individual size (Bélanger-Deschênes et al. 2013; Jacobsen et al. 2016; Zhang et al. 2016). These results have also been validated in studies of *Sebastiscus marmoratus* (Xu et al. 2017). Currently, *P. minor* has a wide thermal amplitude. After undergoing population expansion, Chinese and Malaysian *P. minor* populations were able to adapt to the habitats of different marine regions and withstand different natural selection pressures. With the passing of time, many new mutations appeared, and abundant haplotype diversity accumulated to form a unique haplotype. Lineage 2 was composed entirely of the haplotype of the Malaysian population, and in lineage 1, only KS appeared in the ancestral haplotype Hap_1, while all other haplotypes were from the Chinese populations. In other words, except for the ancestral haplotype, the remaining haplotypes of the Chinese and Malaysian *P. minor* populations have produced substantial differentiation, each accounting for their unique haplotypes. However, from the current perspective, there was still insufficient time for ample nucleotide variation to be produced. *F*_ST_ results also showed that significant genetic differentiation had occurred between the Chinese and Malaysian *P. minor* populations, further validating their respective accumulation of genetic variation to adapt to their living environments. Due to Malaysia’s low latitude, high water temperature and greater number of habitats, as well as the effect of the monsoon systems, which cause the ocean currents to bring an abundance of plankton as a richer source of food, there is reduced pressure on the Malaysian *P. minor* population, allowing for the accumulation of more genetic variation, which in turn results in higher genetic diversity.

An interesting result was found in the Xiamen population, which showed significant differentiation from other populations, indicating that the breeding patterns of *P. minor* are complex. A second confirmation was performed on the sample sources and the results of the data analysis to eliminate the possible effects of these factors. Studies have reported that when *P. minor* was first discovered, the northern boundary of its distribution range was in the Xiamen marine region, and its geographical distribution range was in the waters south of the Taiwan Strait (Liu and Li 1998). Xiamen is in the northern boundary of the *P. minor* population distribution range; that is, it is a marginal population. In studies of adaptive evolution, the marginal populations of species are more sensitive to environmental changes, exhibit more pronounced population differentiation, and genetic polymorphic sites associated with adaptive evolution are more readily detected (Bridle and Vines 2007). These phenomena have been confirmed in the Xiamen population, and this result, to some extent, also strongly supports the analysis of population genetic patterns and population evolution for marginal effects at a specific spatiotemporal scale of a single species. Xiamen Island is a semi-enclosed island surrounded by the mainland. Freshwater flows from the southeastern part of the Jiulong River and the outside are blocked by Jinmen Island, resulting in complex and variable hydrological environmental factors (Jing et al. 2011). This may also be the cause of *P. minor* differentiation in Xiamen.

Based on these results, we speculate that the time during which *P. minor* expanded from the refugium to occupy the coastal areas of China and Malaysia was relatively short. With the passage of time, the Chinese and Malaysian *P. minor* populations accumulated sufficient genetic variation to diverge completely. Similar results have been detected in the genetic structure of Chinese pomfret with similar distributions. The results of this study on the population genetics of *P. minor* are consistent with the proposed mesoscale boundary units suggested for the management of the region by Ablan et al. (2002). The Chinese coastal population should be classified as a north-central group (encompassing northwestern Taiwan, northern Vietnam, and the northwestern Philippines), and the Malaysian population should be classified as a southwestern group (comprising southern Vietnam and the eastern coast of mainland Malaysia). Unfortunately, the genetic diversity of *P. minor* in coastal China is lower than that of the Malaysian population, which is directly related to the high fishing pressure in China’s offshore waters. Therefore, in order to safeguard fishery resources, emphasis should be placed on the protection of *P. minor* resources, fishing in coastal *P. minor* spawning grounds should be prohibited, and bottom trawling, gillnet and set net operations should be strictly prohibited. If these measures are taken seriously and implemented, *P. minor* resources will gradually recover, and the tragedy of resource decline of traditional commercial fishes in China, such as the large yellow croaker (*Larimichthys crocea*), small yellow croaker (*Larimichthys polyactis*), and hairtail (*Trichiurus haumela*), will been avoided.

## Conclusion

Genetic signature of *P. minor* in China and Malaysia coastal waters were evaluated. The results showed that all *P. minor* had moderate haplotype diversity and two divergent lineages. The phylogenetic structure of *P. minor* corresponded imperfectly with geographical location at the Cytb gene level, but significant divergence between Chinese and Malaysian populations was detected. To get precise phylogeographic structure, more sensitive DNA markers such as SLAF, RAD and WGS will be employed and reveal the adaptive evolution mechanism of this species. Lower haplotype diversity is detected in China, which further indicated that Chinese fishery resources are facing greater fishing pressure and more focus is needed on fishery protection and management.
